# Supplementary material for: PolyA tail segmentation improves the stability of the template DNA and increases the translatability of in vitro transcribed mRNA
Source: Nucleic Acids Res. 2026 Jan 15;54(2):gkaf1412. doi: 10.1093/nar/gkaf1412 (PMC12805897; doi:10.1093/nar/gkaf1412)
Supplement: gkaf1412_Supplemental_File [file gkaf1412_supplemental_file.pdf]

PolyA tail segmentation improves the stability of the template DNA and increases the translatability of *in vitro* transcribed mRNA

Tomasz Spiewla[1,2], Karol Czubak[3], Zofia Pilch[4], Marek R. Baranowski[1,5], Pawel S. Krawczyk[6], Kamila Affek[6], Wiktor Antczak[6], Marta Szulc-Gasiorowska[2], Sebastian Chmielinski[2], Seweryn Mroczek[6,7], Michal Brouze[8], Dominika Nowis[3], Jakub Golab[4], Andrzej Dziembowski[6,9], Jacek Jemielity[2] & Joanna Kowalska[1,*]

[1] Division of Biophysics, Institute of Experimental Physics, Faculty of Physics, University of Warsaw, Pasteura 5, 02-093 Warsaw, Poland

[2] Centre of New Technologies, University of Warsaw, Banacha 2c, 02-097 Warsaw, Poland

[3] Laboratory of Experimental Medicine, Medical University of Warsaw, Nielubowicza 5 Street, 02-097 Warsaw, Poland

[4] Department of Immunology, Medical University of Warsaw, Nielubowicza 5 Street, 02-097 Warsaw, Poland

[5] Explorna Therapeutics sp. z o.o. Zwirki i Wigury 93, 02-089 Warsaw, Poland

[6] Laboratory of RNA Biology, International Institute of Molecular and Cell Biology, Ks. Trojdena 4, 02-106 Warsaw, Poland

[7] Institute of Genetics and Biotechnology, Faculty of Biology, University of Warsaw, Miecznikowa 1, 02-106 Warsaw, Poland

[8] Genome Engineering Facility, International Institute of Molecular and Cell Biology, Ks. Trojdena 4, 02-106 Warsaw, Poland

[9] Department of Embryology, Faculty of Biology, University of Warsaw, Miecznikowa 1, 02-096 Warsaw, Poland

* To whom correspondence should be addressed. Email: jkowalska@fuw.edu.pl

SUPPORTING INFORMATION

Table of contents

Figure S1 Sanger sequencing of modified polyA tail sequences from plasmid DNA encoding FLuc 2

Figure S2 PolyA tail sequence instability in plasmid DNA vectors across bacterial strains. .3

Figure S3 Sequencing reads of DNA Nanopore for selected DNA variants containing modified 3'-tail sequences 4

Figure S4 Sequencing reads of DRS for selected mRNA variants containing modified 3'-tail sequences 5

Figure S5 Dot-blot assay results for dsRNA detection in mRNA samples with modified 3'-terminal sequences 6

Figure S6 Analysis of mRNA purity and protein expression for variants containing 1-methyl-pseudouridine (m1Ψ) and uridine (U)....... 7

Figure S7 Comparison of FLuc protein expression levels *in vitro* from mRNAs bearing short (A_{30}) or long (A_{90}) polyA tails 8

Figure S8 Cell viability following transfection with FLuc mRNAs containing different modified 3'-tail sequences 9

Figure S9 Quality control of lipid nanoparticles preparation for *in vivo* experiments. mRNA variants of hEPO.. 10

Figure S10 Schematic representation of *in vivo* experiments assessing protein expression levels following LNP-mRNA administration 11

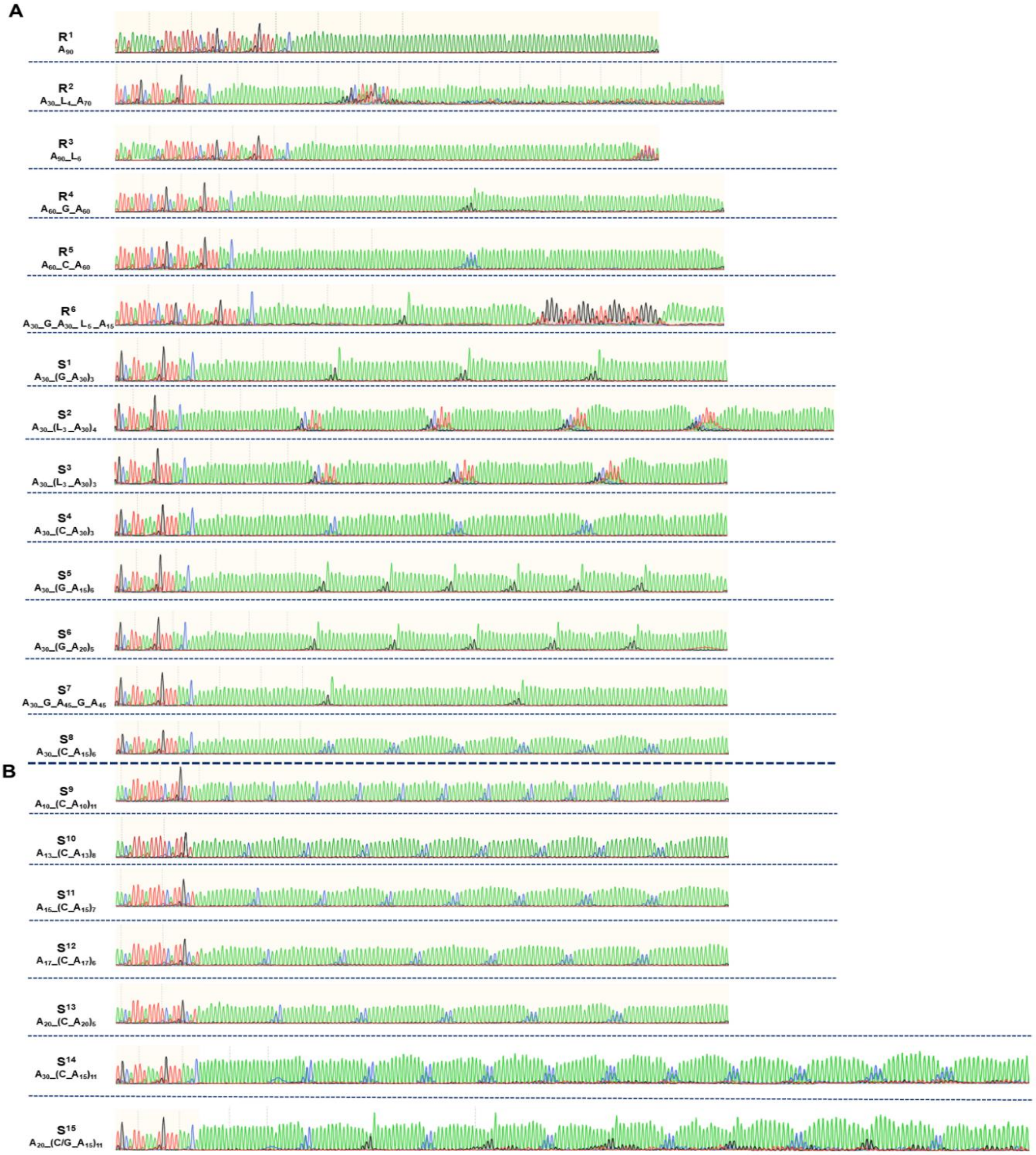

Figure S1 Sanger sequencing of modified polyA tail sequences from plasmid DNA encoding FLuc. The sequencing reads depict the profiles of analysed polyA tail variants, which include nucleotide modifications. Variants R^1–R^6 represent reference polyA tail sequences, while S^1–S^{15} contain various insertions and modifications. Below the modification names, the corresponding summary formulas of the modifications are provided. The coloured peaks correspond to nucleotide bases: adenine (green), cytosine (blue), guanine (black), and thymine (red).

A

B

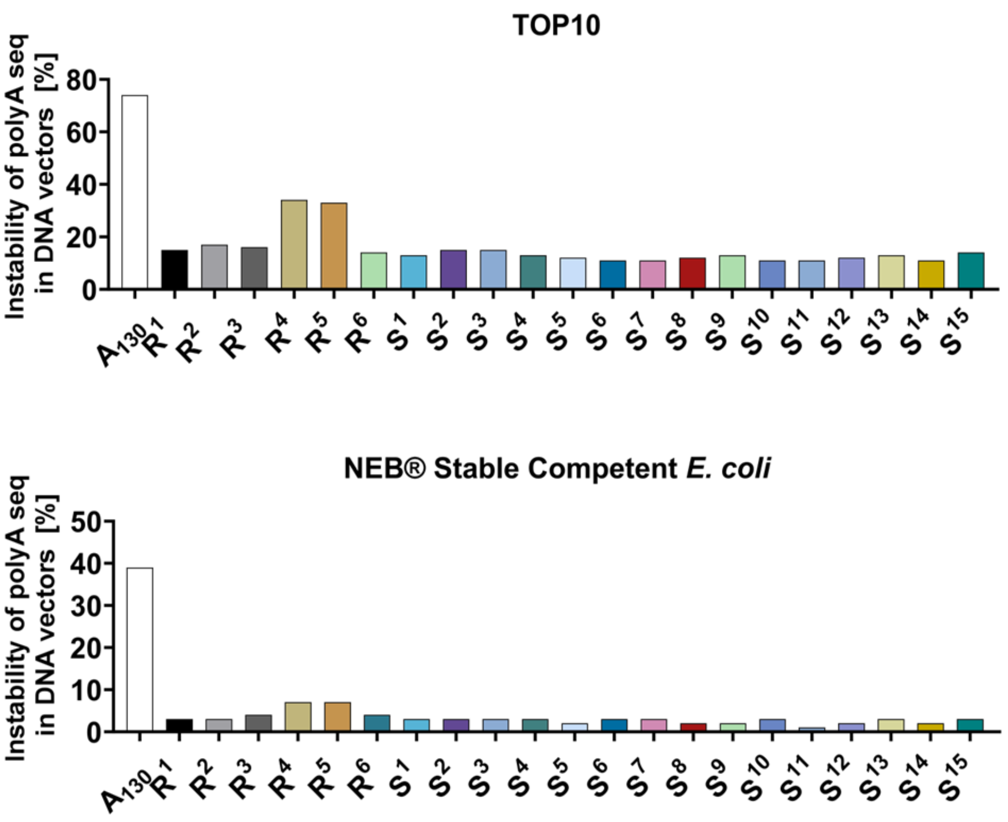

Figure S2 PolyA tail sequence instability in plasmid DNA vectors across bacterial strains. Instability levels of FLuc mRNA polyA variants (R^1-R^6, S^1-S^{15}) in TOP10 and NEB® Stable Competent *E. coli* are shown as the percentage of clones with altered polyA tail length or sequence relative to the original construct.

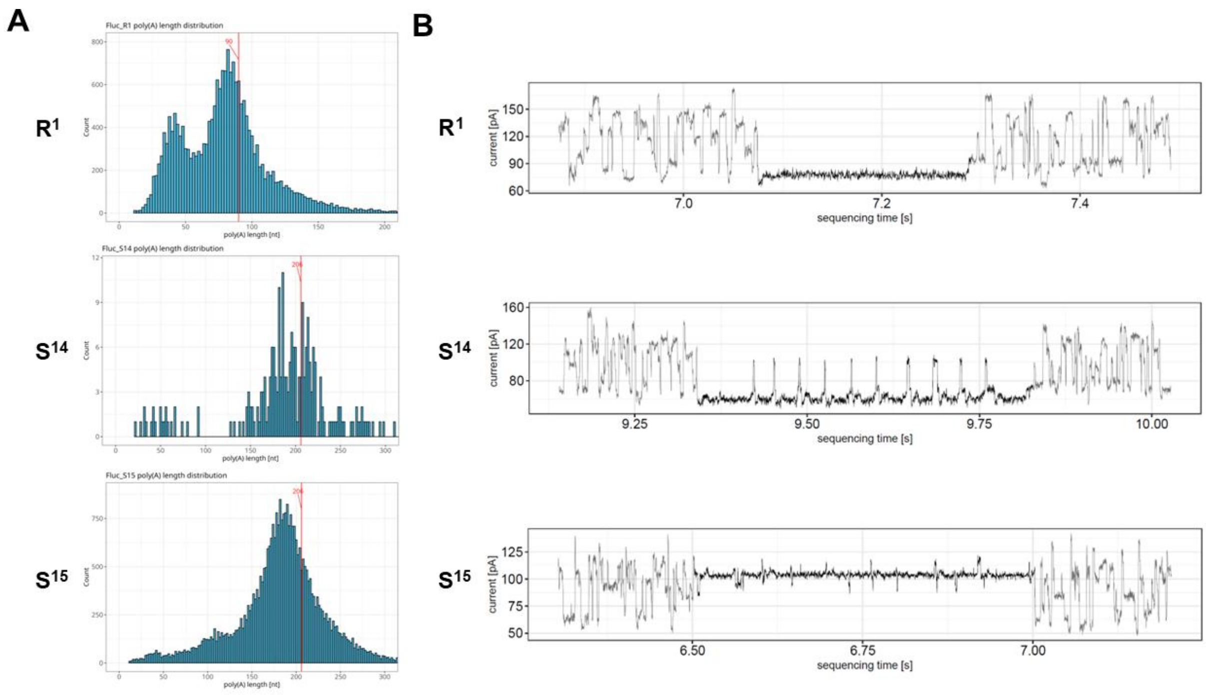

Figure S3 Sequencing reads of DNA Nanopore for selected DNA variants containing modified 3'-tail sequences. A) Histograms of polyA tail length determined by DNA Nanopore sequencing for selected DNA variants (R^1, S^{14} and S^{15}). B) Changes in current intensity over time for different polyA tail variants with incorporated heteronucleotides. The stepwise changes in intensity reflect the presence of heteronucleotides in the polyA tail structure, which enabled the precise identification of linker types and lengths in the individual DNA variants.

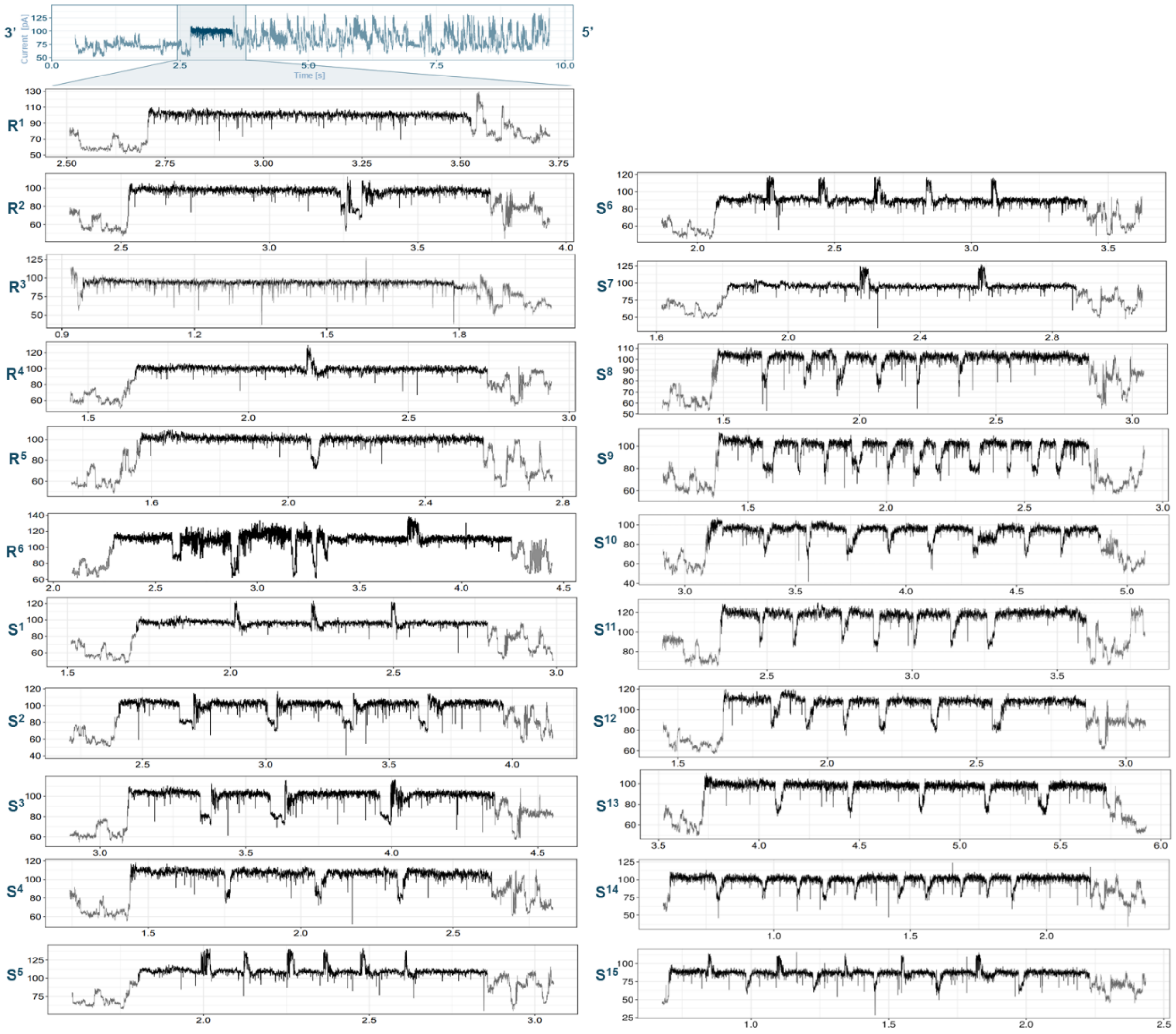

Figure S4 Sequencing reads of DRS for selected mRNA variants containing modified 3'-tail sequences. Each graph shows changes in current intensity over time for different polyA tail variants with incorporated heteronucleotides. The stepwise changes in intensity reflect the presence of heteronucleotides in the polyA tail structure, which enabled the precise identification of linker types and lengths in the individual mRNA variants.

Dot-blot membrane for mRNA with modified 3' terminal sequences

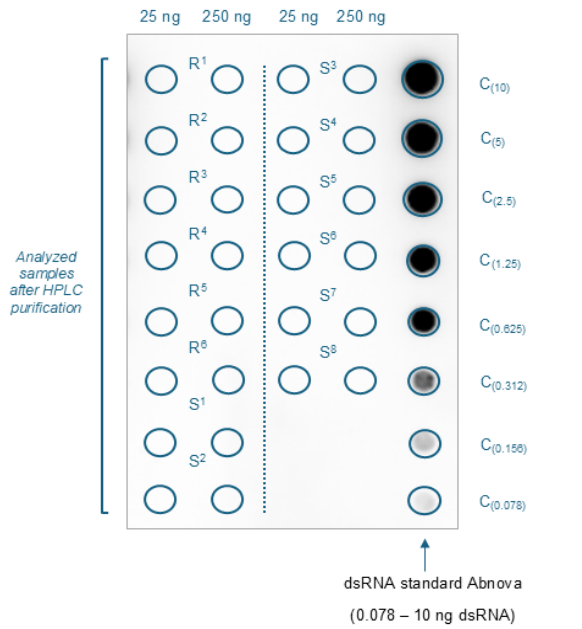

n.d. – (not detected) dsRNA is not present in the analyzed sample or dsRNA content is below the detection limit of this method

**<0.078 ng dsRNA/25 ng of blotted mRNA (<3.12‰)*

***<0.078 ng dsRNA/250 ng of blotted mRNA (<0.31‰)*

dsRNA content analyzed in mRNA samples after HPLC purification

mRNA ID	25 ng mRNA	250 ng mRNA
R^1	n.d.<br>*<3.12‰	n.d.<br>**<0.31‰
R^2	n.d.<br>*<3.12‰	n.d.<br>**<0.31‰
R^3	n.d.<br>*<3.12‰	n.d.<br>**<0.31‰
R^4	n.d.<br>*<3.12‰	n.d.<br>**<0.31‰
R^5	n.d.<br>*<3.12‰	n.d.<br>**<0.31‰
R^6	n.d.<br>*<3.12‰	n.d.<br>**<0.31‰
S^1	n.d.<br>*<3.12‰	n.d.<br>**<0.31‰
S^2	n.d.<br>*<3.12‰	n.d.<br>**<0.31‰
S^3	n.d.<br>*<3.12‰	n.d.<br>**<0.31‰
S^4	n.d.<br>*<3.12‰	n.d.<br>**<0.31‰
S^5	n.d.<br>*<3.12‰	n.d.<br>**<0.31‰
S^6	n.d.<br>*<3.12‰	n.d.<br>**<0.31‰
S^7	n.d.<br>*<3.12‰	n.d.<br>**<0.31‰
S^8	n.d.<br>*<3.12‰	n.d.<br>**<0.31‰

Figure S5 Dot-blot assay results for dsRNA detection in mRNA samples with modified 3'-terminal sequences. Reference variants are labeled as R^1–R^6, while modified variants are designated as S^1–S^8. dsRNA levels were determined based on the standard curve range for dsRNA (0.078–10 ng dsRNA). The table provides quantitative results for samples containing 25 ng and 250 ng of mRNA, detailing detection limits (<0.078 ng dsRNA) and presenting results as a percentage of the total mRNA mass.

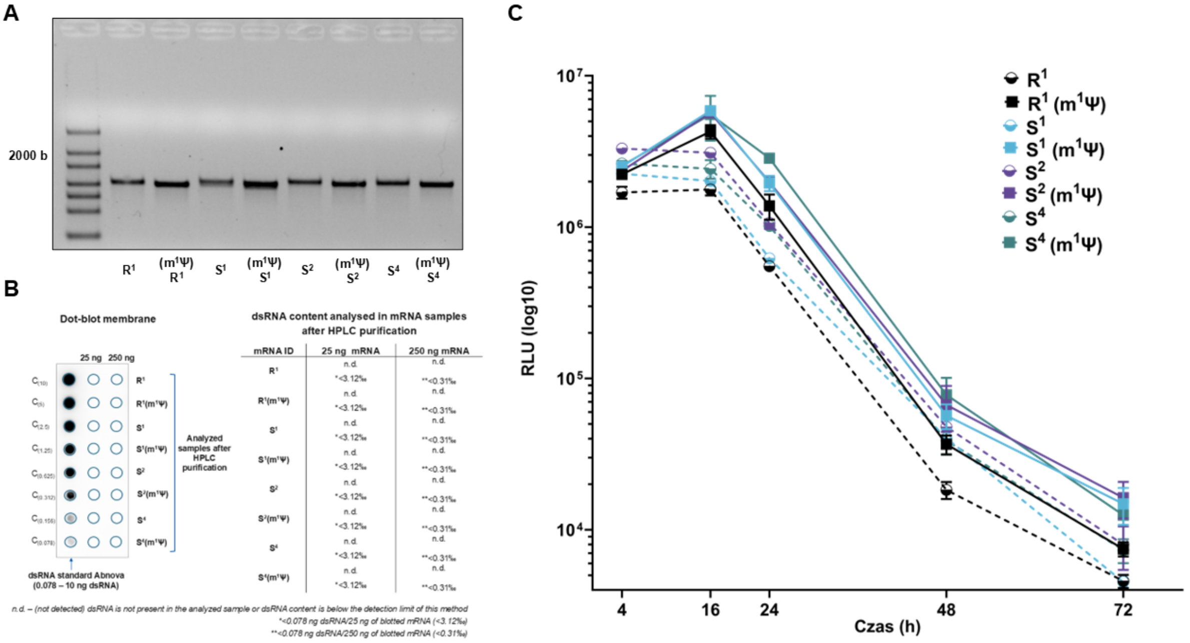

Figure S6 Analysis of mRNA purity and protein expression for variants containing 1-methyl-pseudouridine (m1Ψ) and uridine (U). A) Electropherogram of purified mRNA samples, demonstrating high homogeneity and successful incorporation of m1Ψ. The observed shift in RNA bands for m1Ψ-modified samples, compared to their U-containing counterparts, confirms the presence of the modification. B) Dot-blot dsRNA analysis of mRNA samples after HPLC purification, showed no detectable levels of double-stranded RNA (C) FLuc protein expression kinetics in HEK293T cells, measured in relative light units (RLU) at defined time points (4 h, 16 h, 24 h, 48 h, and 72 h).

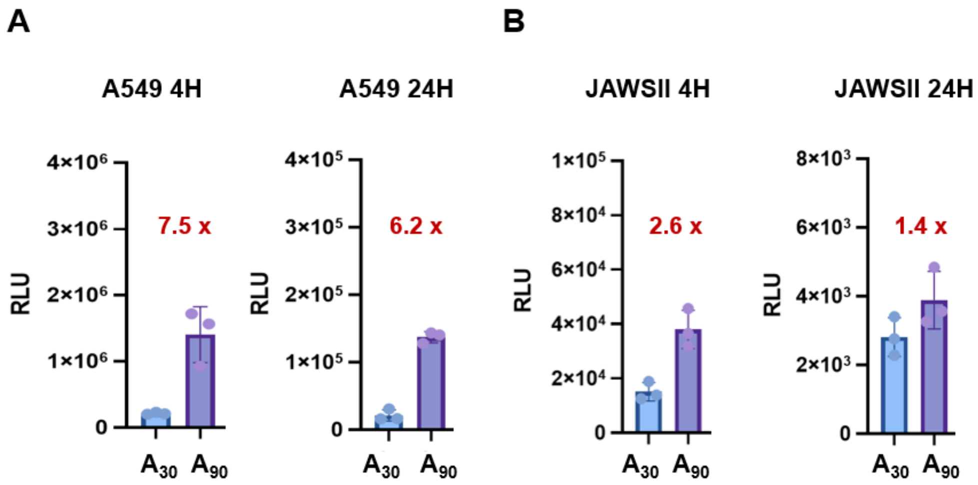

Figure S7 Comparison of FLuc protein expression levels *in vitro* from mRNAs bearing short (A_{30}) or long (A_{90}) polyA tails. A) FLuc expression in A549 cells at 4 and 24 hours post-transfection. A substantial increase in expression was observed for the A_{90} variant, as indicated in red. B) FLuc expression in JAWSII cells at 4 and 24 hours post-transfection. The A_{90} variant consistently outperformed A_{30}, although the difference was less pronounced than in A549 cells. Fold changes in red denote the relative increase in expression from A_{90} mRNA compared to A_{30}.

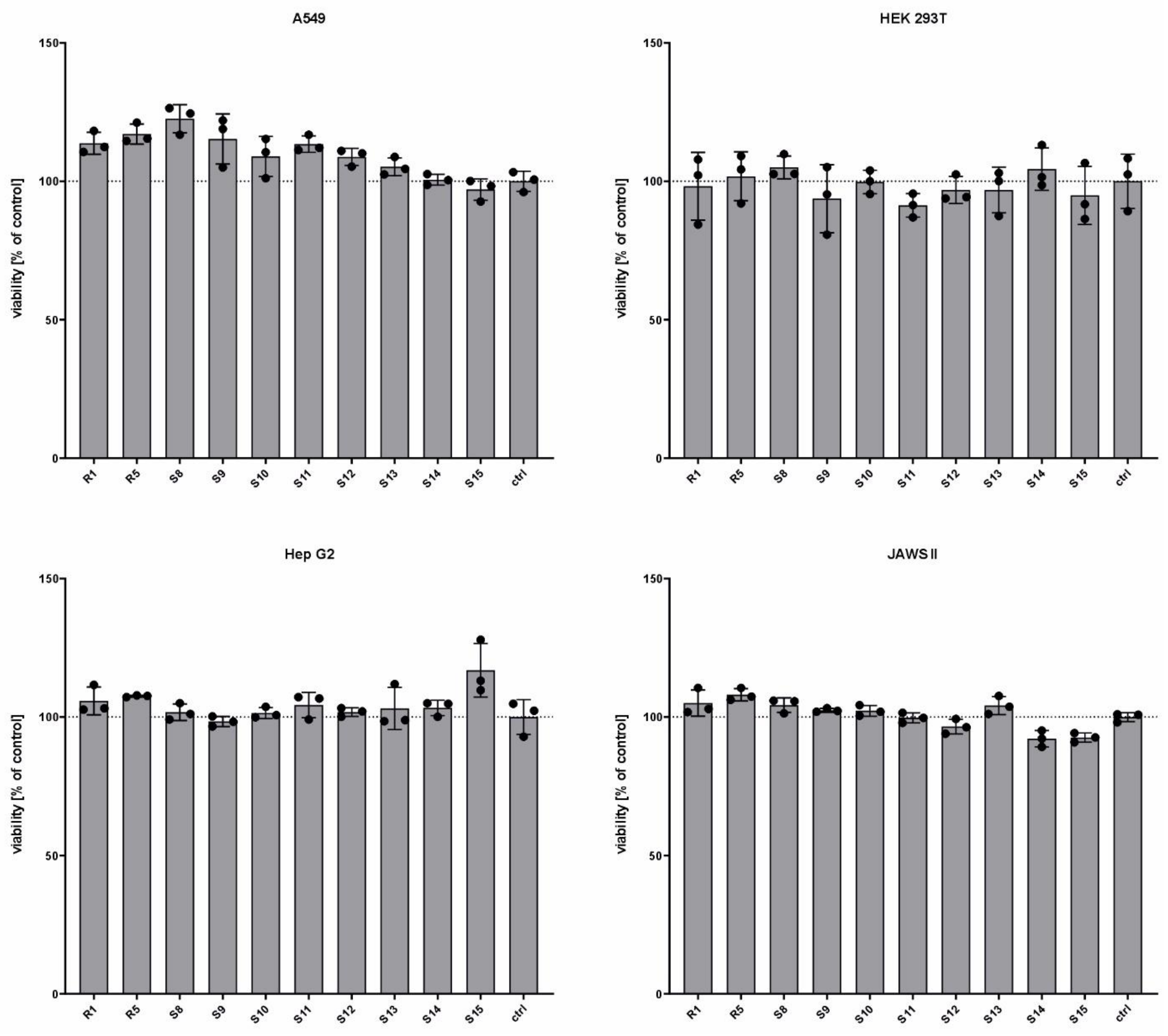

Figure S8 Cell viability following transfection with FLuc mRNAs containing different modified 3'-tail sequences. Viability of A549, HEK 293T, Hep G2, and JAWS II cells was assessed 24 h post-transfection using Cell Titer Blue assay (Promega). Bars represent viability relative to untreated control cells; means ± SD; n = 3.

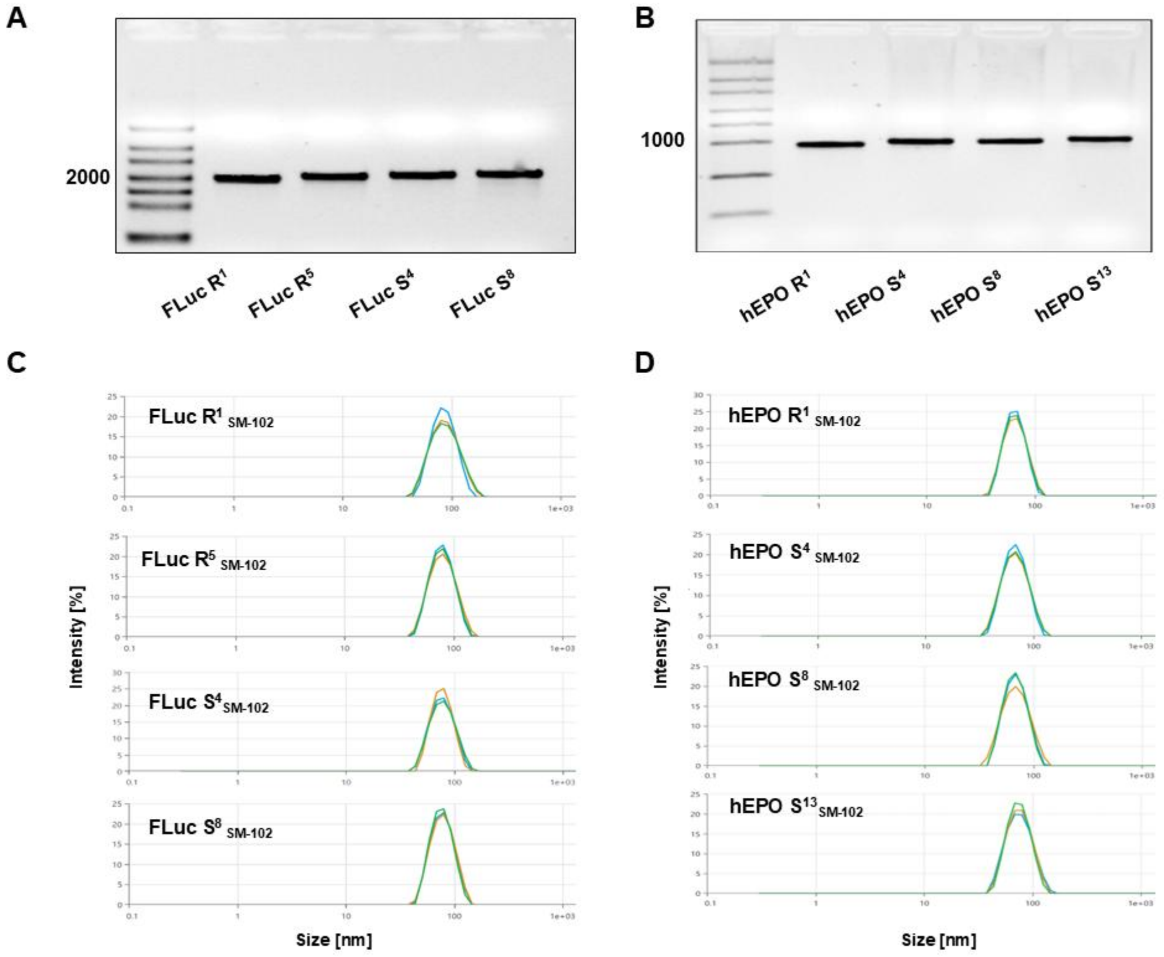

Figure S9 Quality control of lipid nanoparticles preparation for *in vivo* experiments. A) Electropherograms of all tested mRNA variants, encoding Firefly luciferase (FLuc R^1 - FLuc S^8) and B) human erythropoietin (hEPO R^1 - hEPO S^{13}). C) Quality and homogeneity assessment of formulated LNPs (SM-102 lipid mix) for all mRNA variants of FLuc and D) all mRNA variants of hEPO. The graphs illustrate intensity fluctuations over time, determining nanoparticle size and uniformity.

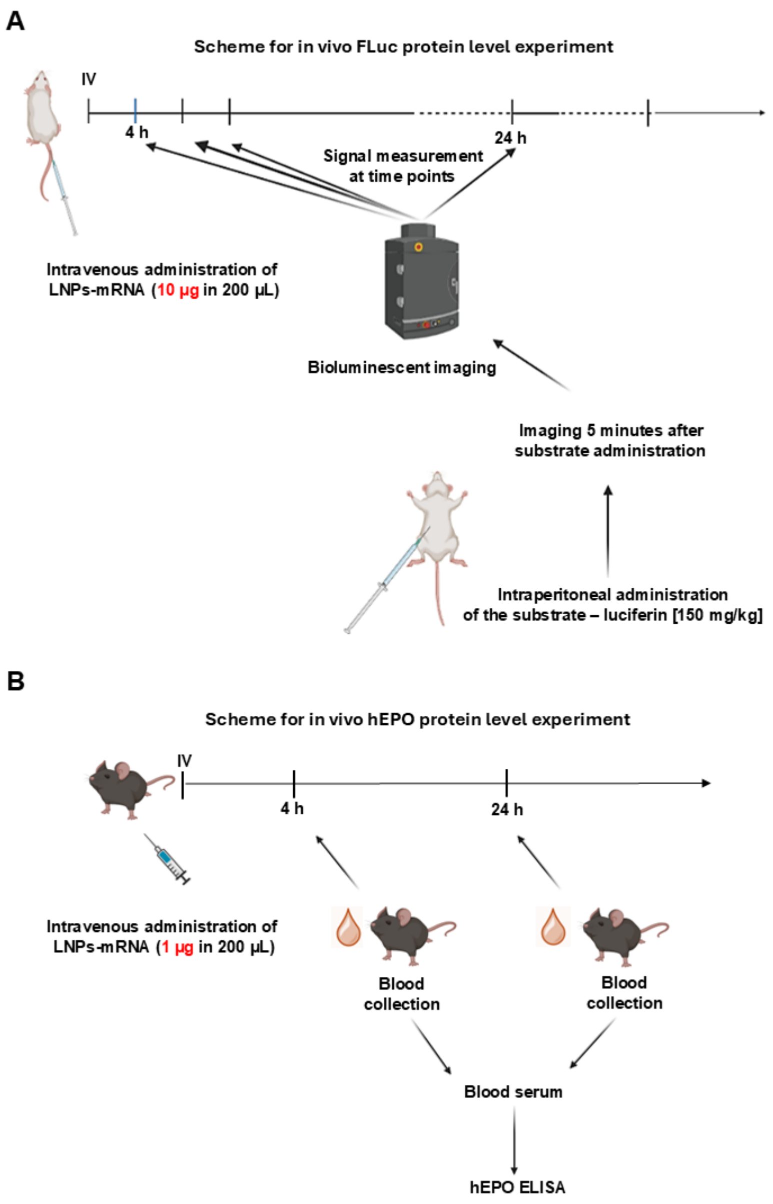

Figure S10 Schematic representation of *in vivo* experiments assessing protein expression levels following LNP-mRNA administration. A) Assessment of *in vivo* FLuc protein expression using bioluminescence imaging. To evaluate the expression of Firefly luciferase (FLuc) *in vivo*, mice received an intravenous (IV) injection of LNP-mRNA (10 µg in 200 µL). Bioluminescence imaging was performed at multiple time points, including 4 h and 24 h post-injection. Five minutes before imaging, luciferin substrate (150 mg/kg) was administered intraperitoneally (IP) to enable bioluminescent signal detection. B) Evaluation of *in vivo* hEPO protein levels through blood serum analysis. To assess the expression of human erythropoietin (hEPO) *in vivo*, mice received an IV injection of LNP-mRNA (1 µg in 200 µL). Blood samples were collected at 4 h and 24 h post-injection to monitor protein levels over time. Serum was then isolated for hEPO quantification using an ELISA assay.

Table S1. Summary of complementary DNA oligonucleotide sequences used for the preparation of DNA plasmid templates with modified polyA sequences

Mod		DNA oligonucleotide sequence
**R[1]**	Starter F	AAAAAAAAAAAAAAAAAAAAAAAAAAAAAAAAAAAAAAAAAAAAAAAAAAAAAAAAAAAAAA
	Starter R	TTTTTTTTTTTTTTTTTTTTTTTTTTTTTTTTTTTTTTTTTTTTTTTTTTTTTTTTTTTTTT
**R[2]**	Starter F	AAAAAAAAAAAAAAAAAAAAAAAAAAAAAAAAGCATATGACTAAAAAAAAAAAAAAAAAAAAAAAAAAAA<br>AAAAAAAAAAAAAAAAAAAAAAAAAAAAAAAAAAAAAAAAAAAA
	Starter R	TTTTTTTTTTTTTTTTTTTTTTTTTTTTTTTTTTTTTTTTTTTTTTTTTTTTTTTTTTTTTTTTTTTTTT<br>TTAGTCATATGCTTTTTTTTTTTTTTTTTTTTTTTTTTTTTTTT
**R[3]**	Starter F	AAAAAAAAAAAAAAAAAAAAAAAAAAAAAAAAAAAAAAAAAAAAAAAAAAAAAAAAATCTAG
	Starter R	CTAGATTTTTTTTTTTTTTTTTTTTTTTTTTTTTTTTTTTTTTTTTTTTTTTTTTTTTTTTT
**R[4]**	Starter F	AAAAAAAAAAAAAAAAAAAAAAAAAAAAAAAAGAAAAAAAAAAAAAAAAAAAAAAAAAAAAAAAAAAAAA<br>AAAAAAAAAAAAAAAAAAAAAAA
	Starter R	TTTTTTTTTTTTTTTTTTTTTTTTTTTTTTTTTTTTTTTTTTTTTTTTTTTTTTTTTTTTCTTTTTTTTT<br>TTTTTTTTTTTTTTTTTTTTTTT
**R[5]**	Starter F	AAAAAAAAAAAAAAAAAAAAAAAAAAAAAAAACAAAAAAAAAAAAAAAAAAAAAAAAAAAAAAAAAAAAA<br>AAAAAAAAAAAAAAAAAAAAAAA
	Starter R	TTTTTTTTTTTTTTTTTTTTTTTTTTTTTTTTTTTTTTTTTTTTTTTTTTTTTTTTTTTTGTTTTTTTTT<br>TTTTTTTTTTTTTTTTTTTTTT
**R[6]**	Starter F	AAGAAAAAAAAAAAAAAAAAAAAAAAAAAAAAATGGGGTT<br>TGGGGTTTGGGGTTTGGGGTAAAAAAAAAAAAAAA
	Starter R	TTTTTTTTTTTTTTTACCCCAAACCCCAAACCCCAAACCCCATTTTTTTTTTTTTTTTTTTTTTTTTTTT<br>TTCTT
**S[1]**	Starter F	AAGAAAAAAAAAAAAAAAAAAAAAAAAAAAAAAGAAAAAAAAAAAAAAAAAAAAAAAAAAAAAAGAAAAA<br>AAAAAAAAAAAAAAAAAAAAAAAAA
	Starter R	TTTTTTTTTTTTTTTTTTTTTTTTTTTTTTCTTTTTTTTTTTTTTTTTTTTTTTTTTTTTTCTTTTTTTT<br>TTTTTTTTTTTTTTTTTTTTTTCTT
**S[2]**	Starter F	AAGCATATAAAAAAAAAAAAAAAAAAAAAAAAAAAAAAGCATATAAAAAAAAAAAAAAAAAAAAAAAAAA<br>AAAAGCATATAAAAAAAAAAAAAAAAAAAAAAAAAAAAAAGCATA<br>TAAAAAAAAAAAAAAAAAAAAAAAAAAAAAA
	Starter R	TTTTTTTTTTTTTTTTTTTTTTTTTTTTTTATATGCTTTTTTTTTTTTTTTTTTTTTTTTTTTTTTATAT<br>GCTTTTTTTTTTTTTTTTTTTTTTTTTTTTTTATATGCTTTTTTTTTTTTTTTTTTTTTTTTTTTTTTAT<br>ATGCTT
**S[3]**	Starter F	AAGCATATAAAAAAAAAAAAAAAAAAAAAAAAAAAAAAGCATATAAAAAAAAAAAAAAAAAAAAAAAAAA<br>AAAAGCATATAAAAAAAAAAAAAAAAAAAAAAAAAAAAAA
	Starter R	TTTTTTTTTTTTTTTTTTTTTTTTTTTTTTATATGCTTTTTTTTTTTTTTTTTTTTTTTTTTTTTTATAT<br>GCTTTTTTTTTTTTTTTTTTTTTTTTTTTTTTATATGCTT
**S[4]**	Starter F	AACAAAAAAAAAAAAAAAAAAAAAAAAAAAAAACAAAAAAAAAAAAAAAAAAAAAAAAAAAAAACAAAAA<br>AAAAAAAAAAAAAAAAAAAAAAAAA
	Starter R	TTTTTTTTTTTTTTTTTTTTTTTTTTTTTTGTTTTTTTTTTTTTTTTTTTTTTTTTTTTTTGTTTTTTTT<br>TTTTTTTTTTTTTTTTTTTTTTGTT
**S[5]**	Starter F	AAGAAAAAAAAAAAAAAAGAAAAAAAAAAAAAAAGAAAAAAAAAAAAAAAGAAAAAAAAAAAAAAAGAAA<br>AAAAAAAAAAAAGAAAAAAAAAAAAAAA
	Starter R	TTTTTTTTTTTTTTTCTTTTTTTTTTTTTTTCTTTTTTTTTTTTTTTCTTTTTTTTTTTTTTTCTTTTTT<br>TTTTTTTTTCTTTTTTTTTTTTTTTCTT
**S[6]**	Starter F	AAGAAAAAAAAAAAAAAAAAAAAGAAAAAAAAAAAAAAAA<br>AAAAGAAAAAAAAAAAAAAAAAAAAGAAAAAAAAAAAAAA AAAAAAGAAAAAAAAAAAAAAAAAAAA
	Starter R	TTTTTTTTTTTTTTTTTTTTCTTTTTTTTTTTTTTTTTTTTCTTTTTTTTTTTTTTTTTTTTCTTTTTTT<br>TTTTTTTTTTTTTCTTTTTTTTTTTTTTTTTTTTCTT
**S[7]**	Starter F	AAGAAAAAAAAAAAAAAAAAAAAAAAAAAAAAAAAAAAAAAAAAAAAAGAAAAAAAAAAAAAAAAAAAAA<br>AAAAAAAAAAAAAAAAAAAAAAAA
	Starter R	TTTTTTTTTTTTTTTTTTTTTTTTTTTTTTTTTTTTTTTTTTTTTCTTTTTTTTTTTTTTTTTTTTTTTT<br>TTTTTTTTTTTTTTTTTTTTTCTT
**S[8]**	Starter F	AACAAAAAAAAAAAAAAACAAAAAAAAAAAAAAACAAAAAAAAAAAAAAACAAAAAAAAAAAAAAACAAA<br>AAAAAAAAAAAACAAAAAAAAAAAAAAA
	Starter R	TTTTTTTTTTTTTTTGTTTTTTTTTTTTTTTGTTTTTTTTTTTTTTTGTTTTTTTTTTTTTTTGTTTTTT<br>TTTTTTTTTGTTTTTTTTTTTTTTTGTT
**S[9]**	Starter F	TAAAAAAAAAACAAAAAAAAAACAAAAAAAAAACAAAAAAAAAACAAAAAAAAAACAAAAAAAAAACAAA<br>AAAAAAACAAAAAAAAAACAAAAAAAAAACAAAAAAAAAACAAAAAAAAAACAAAAAAAAAA
	Starter R	TTTTTTTTTTGTTTTTTTTTTGTTTTTTTTTTGTTTTTTTTTTGTTTTTTTTTTGTTTTTTTTTTGTTTT<br>TTTTTTGTTTTTTTTTTGTTTTTTTTTTGTTTTTTTTTTGTTTTTTTTTTGTTTTTTTTTTA
**S[10]**	Starter F	TAAAAAAAAAAAAACAAAAAAAAAAAAACAAAAAAAAAAAAAAACAAAAAAAAAAAAACAAAAAAAAAAAAA<br>CAAAAAAAAAAAAACAAAAAAAAAAAAACAAAAAAAAAAAAACAAAAAAAAAAAAA

	Starter R	TTTTTTTTTTTTTGTTTTTTTTTTTTTGTTTTTTTTTTTTTGTTTTTTTTTTTTTGTTTTTTTTTTTTTGTTTTTTTTTTTTTGTTTTTTTTTTTTTGTTTTTTTTTTTTTGTTTTTTTTTTTTTA
**S11**	Starter F	TAAAAAAAAAAAAAAACAAAAAAAAAAAAAAACAAAAAAAAAAAAAAACAAAAAAAAAAAAAAACAAAAAAAAAAAAAAACAAAAAAAAAAAAAAACAAAAAAAAAAAAAAACAAAAAAAAAAAAAAA
	Starter R	TTTTTTTTTTTTTTTGTTTTTTTTTTTTTTTGTTTTTTTTTTTTTTTGTTTTTTTTTTTTTTTGTTTTTTTTTTTTTTTGTTTTTTTTTTTTTTTGTTTTTTTTTTTTTTTGTTTTTTTTTTTTTTTA
**S12**	Starter F	TAAAAAAAAAAAAAAAAACAAAAAAAAAAAAAAAAACAAAAAAAAAAAAAAAAACAAAAAAAAAAAAAAAAACAAAAAAAAAAAAAAAAACAAAAAAAAAAAAAAAAACAAAAAAAAAAAAAAAAA
	Starter R	TTTTTTTTTTTTTTTTTGTTTTTTTTTTTTTTTTTGTTTTTTTTTTTTTTTTTGTTTTTTTTTTTTTTTTTGTTTTTTTTTTTTTTTTTGTTTTTTTTTTTTTTTTTGTTTTTTTTTTTTTTTTTA
**S13**	Starter F	TAAAAAAAAAAAAAAAAAAAACAAAAAAAAAAAAAAAAAAAACAAAAAAAAAAAAAAAAAAAACAAAAAAAAAAAAAAAAAAAACAAAAAAAAAAAAAAAAAAAACAAAAAAAAAAAAAAAAAAAA
	Starter R	TTTTTTTTTTTTTTTTTTTTGTTTTTTTTTTTTTTTTTTTTGTTTTTTTTTTTTTTTTTTTTGTTTTTTTTTTTTTTTTTTTTGTTTTTTTTTTTTTTTTTTTTGTTTTTTTTTTTTTTTTTTTTA
**S14**	Starter F	AACAAAAAAAAAAAAAAACAAAAAAAAAAAAAAACAAAAAAAAAAAAAAACAAAAAAAAAAAAAAACAAAAAAAAAAAAAAACAAAAAAAAAAAAAAACAAAAAAAAAAAAAAACAAAAAAAAAAAAAAACAAAAAAAAAAAAAAACAAAAAAAAAAAAAAA
	Starter R	TTTTTTTTTTTTTTTGTTTTTTTTTTTTTTTGTTTTTTTTTTTTTTTGTTTTTTTTTTTTTTTGTTTTTTTTTTTTTTTGTTTTTTTTTTTTTTTGTTTTTTTTTTTTTTTGTTTTTTTTTTTTTTTGTTTTTTTTTTTTTTTGTTTTTTTTTTTTTTTGTT
**S15**	Starter F	AACAAAAAAAAAAAAAAAGAAAAAAAAAAAAAAACAAAAAAAAAAAAAAAGAAAAAAAAAAAAAAACAAAAAAAAAAAAAAAGAAAAAAAAAAAAAAACAAAAAAAAAAAAAAAGAAAAAAAAAAAAAAACAAAAAAAAAAAAAAAGAAAAAAAAAAAAAAA
	Starter R	TTTTTTTTTTTTTTTCTTTTTTTTTTTTTTTGTTTTTTTTTTTTTTTCTTTTTTTTTTTTTTTGTTTTTTTTTTTTTTTCTTTTTTTTTTTTTTTGTTTTTTTTTTTTTTTCTTTTTTTTTTTTTTTGTTTTTTTTTTTTTTTCTTTTTTTTTTTTTTTGTT

Table S2. Coding DNA sequences for the Firefly luciferase, mKate2_PEST, human erythropoietin proteins, and human alpha-1 antitrypsin used as reporter genes in mRNA studies with modified 3'-tail sequences.

DNA Sequence Name	DNA Sequence
Firefly Lucyferase (FLuc)	ATGGAAGACGCCAAAAACATAAAGAAAGGCCCGGCGCCATTCTATCCTCTAGAGGATGGAACCGCTGGAGAGCAA<br>CTGCATAAGGCTATGAAGAGATACGCCCTGGTTCCTGGAACAATTGCTTTTACAGATGCACATATCGAGGTGAAC<br>ATCACGTACGCGGAATACTTCGAAATGTCCGTTCGGTTGGCAGAAGCTATGAAACGATATGGGCTGAATACAAAT<br>CACAGAATCGTCGTATGCAGTGAAAACTCTCTTCAATTCTTTATGCCGGTGTTGGGCGCGTTATTTATCGGAGTT<br>GCAGTTGCGCCCGCGAACGACATTTATAATGAACGTGAATTGCTCAACAGTATGAACATTTCGCAGCCTACCGTA<br>GTGTTTGTTTCCAAAAAGGGGTTGCAAAAAATTTTGAACGTGCAAAAAAAATTACCAATAATCCAGAAAATTATT<br>ATCATGGATTCTAAAACGGATTACCAGGGATTTCAGTCGATGTACACGTTCGTCACATCTCATCTACCTCCCGGT<br>TTTAATGAATACGATTTTGTACCAGAGTCCTTTGATCGTGACAAAACAATTGCACTGATAATGAATTCCTCTGGA<br>TCTACTGGGTTACCTAAGGGTGTGGCCCTTCCGCATAGAACTGCCTGCGTCAGATTCTCGCATGCCAGAGATCCT<br>ATTTTTGGCAATCAAATCATTCCGGATACTGCGATTTTAAGTGTTGTTCCATTCCATCACGGTTTTGGAATGTTT<br>ACTACACTCGGATATTTGATATGTGGATTTCGAGTCGTCTTAATGTATAGATTTGAAGAAGAGCTGTTTTTACGA<br>TCCCTTCAGGATTACAAAATTCAAAGTGCGTTGCTAGTACCAACCCTATTTTCATTCTTCGCCAAAAGCACTCTG<br>ATTGACAAATACGATTTATCTAATTTACACGAAATTGCTTCTGGGGGCGCACCTCTTTCGAAAGAAGTCGGGGAA<br>GCGGTTGCAAAACGCTTCCATCTTCCAGGGATACGACAAGGATATGGGCTCACTGAGACTACATCAGCTATTCTG<br>ATTACACCCGAGGGGGATGATAAACCGGGCGCGGTCGGTAAAGTTGTTCCATTTTTTGAAGCGAAGGTTGTGGAT<br>CTGGATACCGGGAAAACGCTGGGCGTTAATCAGAGAGGCGAATTATGTGTCAGAGGACCTATGATTATGTCCGGT<br>TATGTAAACAATCCGGAAGCGACCAACGCCTTGATTGACAAGGATGGATGGCTACATTCTGGAGACATAGCTTAC<br>TGGGACGAAGACGAACACTTCTTCATAGTTGACCGCTTGAAGTCTTTAATTAAATACAAAGGATATCAGGTGGCC<br>CCCGCTGAATTGGAATCGATATTGTTACAACACCCCAACATCTTCGACGCGGGCGTGGCAGGTCTTCCCGACGAT<br>GACGCCGGTGAACTTCCCGCCGCCGTTGTTGTTTTGGAGCACGGAAAGACGATGACGGAAAAAGAGATCGTGGAT<br>TACGTCGCCAGTCAAGTAACAACCGCGAAAAAGTTGCGCGGAGGAGTTGTGTTTGTGGACGAAGTACCGAAAGGT<br>CTTACCGGAAAACTCGACGCAAGAAAAATCAGAGAGATCCTCATAAAGGCCAAGAAGGGCGGAAAGTCCAAATTG<br>TAA
mKate2-PEST	ATGGTCAGCGAGTTGATCAAGGAGAACATGCACATGAAGCTGTATATGGAGGGGACCGTCAACAACCACCACTTC<br>AAATGCACGTCCGAGGGGGAGGGCAAACCGTACGAGGGCACCCAGACGATGCGGATCAAGGCCGTCGAGGGGGGC<br>CCtCTCCCCTTCGCCTTCGATATCCTCGCGACCAGCTTCATGTACGGCTCCAAGACGTTCATCAACCACACGCAG<br>GGCATCCCAGACTTCTTCAAGCAGTCGTTCCCGGAAGGGTTCACGTGGGAGAGGGTGACCACGTACGAAGACGGT<br>GGGGTGTTGACCGCTACGCAAGACACGTCCCTCCAGGACGGCTGCCTGATTTACAACGTCAAGATCCGGGGCGTC<br>AACTTCCCGAGCAACGGGCCCGTAATGCAGAAGAAGACTTTGGGGTGGGAGGCCTCGACCGAGACCTTGTACCCC<br>GCCGACGGCGGCCTTGAGGGGCGAGCTGACATGGCTCTCAAGCTCGTCGGCGGGGGACACTTGATCTGCAACCTA<br>AAAACGACGTACAGGTCCAAGAAGCCGGCGAAGAACCTAAAGATGCCTGGCGTCTACTACGTGGACCGGAGGCTC<br>GAGAGGATCAAGGAGGCGGACAAAGAGACCTACGTGGAGCAGCACGAGGTGGCAGTCGCCCGCTACTGCGATCTC<br>CCCAGTAAGCTCGGCCACCGGAGCCACGGATTCCCTCCAGCAGTTGCTGCTCAAGACGACGGGACTCTGCCCATG<br>TCCTGTGCGCAAGAATCTGGAATGGATCGACATCCTGCAGCTTGCGCCAGCGCTAGAATCAACGTGTAA
Human erythropoietin (hEPO)	ATGGGCGTGCACGAGTGCCCCGCCTGGCTGTGGCTGCTGCTGAGCCTGCTGAGCCTGCCCCTGGGCCTGCCCGTG<br>CTGGGCGCCCCCCCCCGGCTGATCTGCGACAGCCGGGTGCTGGAGCGGTACCTGCTGGAGGCCAAGGAGGCCGAG<br>AACATCACCACCGGCTGCGCCGAGCACTGCAGCCTGAACGAGAACATCACCGTGCCCGACACCAAGGTGAACTTC<br>TACGCCTGGAAGCGGATGGAGGTGGGCCAGCAGGCCGTGGAGGTGTGGCAGGGCCTGGCCCTGCTGAGCGAGGCC<br>GTGCTGCGGGGCCAGGCCCTGCTGGTGAACAGCAGCCAGCCCTGGGAGCCCCTGCAGCTGCACGTGGACAAGGCC<br>GTGAGCGGCCTGCGGAGCCTGACCACCCTGCTGCGGGCCCTGGGCGCCCAGAAGGAGGCCATCAGCCCCCCCGAC<br>GCCGCCAGCGCCGCCCCCCTGCGGACCATCACCGCCGACACCTTCCGGAAGCTGTTCCGGGTGTACAGCAACTTC<br>CTGCGGGGCAAGCTGAAGCTGTACACCGGCGAGGCCTGCCGGACCGGCGACCGGTGA
Human alpha-1 antitrypsin (hA1AT)	ATGCCTAGCAGCGTGTCATGGGGTATTCTGCTGCTGGCCGGACTGTGTTGTCTGGTGCCTGTGTCTCTGGCTGAA<br>GATCCTCAAGGCGACGCCGCTCAGAAAACCGATACAAGCCACCACGACCAGGATCACCCCACCTTCAACAAGATC<br>ACCCCTAACCTGGCCGAGTTCGCCTTCAGCCTGTATAGACAGCTGGCCCACCAGAGCAACAGCACCAACATCTTT<br>TTCAGCCCCGTGTCTATCGCCACCGCCTTTGCTATGCTGAGCCTGGGCACAAAGGCCGACACACACGATGAGATC<br>CTGGAAGGCCTGAACTTCAACCTGACAGAGATCCCCGAGGCTCAGATCCACGAGGGCTTTCAAGAGCTGCTGAGA<br>ACCCTGAACCAGCCTGACTCTCAGCTCCAGCTGACAACCGGCAATGGCCTGTTTCTGTCTGAGGGCCTGAAGCTG<br>GTGGACAAGTTCCTGGAAGATGTGAAGAAGCTGTACCACAGCGAGGCCTTCACCGTGAACTTCGGCGATACCGAG<br>GAAGCCAAGAAGCAGATCAACGACTACGTGGAAAAGGGCACCCAGGGCAAGATCGTGGACCTGGTCAAAGAGCTG<br>GACAGAGACACCGTGTTCGCCCTGGTCAACTACATCTTCTTCAAAGGCAAGTGGGAACGCCCCTTCGAAGTGAAG<br>GACACAGAGGAAGAGGACTTCCACGTCGACCAAGTGACCACCGTGAAGGTGCCCATGATGAAGCGGCTGGGCATG<br>TTCAACATCCAGCACTGCAAGAAACTGAGCAGCTGGGTGCTGCTGATGAAGTACCTGGGCAACGCCACAGCCATA<br>TTCTTTCTGCCCGATGAGGGCAAGCTGCAGCACCTGGAAAATGAGCTGACCCACGACATCATCACCAAGTTCCTA<br>GAGAACGAGGACAGAAGAAGCGCCAGCCTGCATCTGCCTAAGCTGAGCATCACCGGCACCTACGATCTGAAGTCT<br>GTGCTGGGACAGCTGGGCATCACAAAGGTGTTCAGCAATGGCGCCGATCTGTCCGGCGTTACAGAAGAGGCTCCT<br>CTGAAGCTGTCCAAGGCCGTGCACAAAGCCGTGCTGACAATCGATGAGAAGGGAACAGAGGCCGCTGGCGCCATG<br>TTTCTGGAAGCTATCCCTATGAGCATCCCGCCTGAAGTGAAGTTCAACAAGCCCTTCGTGTTCCTGATGATCGAA<br>CAGAATACCAAGTCTCCCCTGTTCATGGGCAAAGTGGTCAACCCCACACAGAAATGA

Table S3. mRNA half-lives and translational efficiencies for mKate2_PEST mRNA with modified 3'-tail sequences for A549 and HEK293T cell lines.

Cell line	mRNA variant	mRNA half-life [min]	mRNA half-life SD [min]	mRNA translation rate	mRNA translation rate SD
A549	mKate2_R^{1}	348,93	15,50	0,39	0,04
	mKate2_R^{2}	321,49	7,02	0,47	0,08
	mKate2_R^{3}	406,74	32,07	0,55	0,06
	mKate2_R^{4}	230,43	17,21	0,34	0,08
	mKate2_R^{5}	349,32	66,68	0,54	0,10
	mKate2_R^{6}	41,59	0,00	0,01	0,00
	mKate2_S^{1}	415,08	35,41	0,59	0,07
	mKate2_S^{2}	416,83	40,19	0,41	0,06
	mKate2_S^{3}	347,74	14,74	0,36	0,06
	mKate2_S^{4}	417,24	13,09	0,65	0,03
	mKate2_S^{5}	426,73	8,97	0,71	0,09
	mKate2_S^{6}	455,27	28,46	0,88	0,11
	mKate2_S^{7}	380,67	33,04	0,65	0,06
	mKate2_S^{8}	413,14	28,47	0,77	0,10
293T	mKate2_R^{1}	342,21	24,99	0,97	0,08
	mKate2_R^{2}	345,30	55,08	1,09	0,04
	mKate2_R^{3}	441,80	64,57	1,19	0,13
	mKate2_R^{4}	248,10	20,51	0,68	0,10
	mKate2_R^{5}	399,63	15,29	1,05	0,32
	mKate2_R^{6}	41,59	0,00	0,01	0,01
	mKate2_S^{1}	363,04	13,93	1,22	0,15
	mKate2_S^{2}	339,49	8,07	0,88	0,13
	mKate2_S^{3}	254,31	21,35	0,85	0,12
	mKate2_S^{4}	298,53	14,23	1,15	0,10
	mKate2_S^{5}	296,63	17,42	1,24	0,01
	mKate2_S^{6}	343,43	33,19	1,39	0,17
	mKate2_S^{7}	316,60	17,97	1,19	0,15
	mKate2_S^{8}	303,56	22,67	1,15	0,12
